# GeNemo: a search engine for web-based functional genomic data

**DOI:** 10.1093/nar/gkw299

**Published:** 2016-04-20

**Authors:** Yongqing Zhang, Xiaoyi Cao, Sheng Zhong

**Affiliations:** Department of Bioengineering, University of California San Diego, La Jolla, California 92093, USA

## Abstract

A set of new data types emerged from functional genomic assays, including ChIP-seq, DNase-seq, FAIRE-seq and others. The results are typically stored as genome-wide intensities (WIG/bigWig files) or functional genomic regions (peak/BED files). These data types present new challenges to big data science. Here, we present GeNemo, a web-based search engine for functional genomic data. GeNemo searches user-input data against online functional genomic datasets, including the entire collection of ENCODE and mouse ENCODE datasets. Unlike text-based search engines, GeNemo's searches are based on pattern matching of functional genomic regions. This distinguishes GeNemo from text or DNA sequence searches. The user can input any complete or partial functional genomic dataset, for example, a binding intensity file (bigWig) or a peak file. GeNemo reports any genomic regions, ranging from hundred bases to hundred thousand bases, from any of the online ENCODE datasets that share similar functional (binding, modification, accessibility) patterns. This is enabled by a Markov Chain Monte Carlo-based maximization process, executed on up to 24 parallel computing threads. By clicking on a search result, the user can visually compare her/his data with the found datasets and navigate the identified genomic regions. GeNemo is available at www.genemo.org.

## INTRODUCTION

Functional genomic assays produced new data types. Leveraging DNA sequencing as a high-throughput readout, these assays can interrogate genome-wide distributions of transcription factor binding (ChIP-seq), epigenetic modifications (ChIP-seq), regulatory regions (DNase-seq, FAIRE-seq) and other functional outcomes. The immediate outputs are DNA sequences, which does not become biologically meaningful until being further processed. After processing, the data are typically stored as genome-wide intensities (e.g. bigWig files (1)) or functional regions (peak files (2)). These processed data provide functional information of the genome. The formats of these processed data are very different from those storing DNA sequences (2,3). Thus, functional genomic data bring new computational challenges.

A pressing challenge is to effectively search functional genomic data from online data repositories. To date, the ENCODE and mouse ENCODE projects have released 3312 functional genomic datasets (4–6). There are at least two conceivable means to search these data. One is to ‘search by text’, that is to find relevant words in the data description. It would be straightforward to use Google for such a task. The other is to ‘search inside the functional data’, for example, to search for any binding patterns that are similar to that of a novel transcription factor. To better appreciate the difference of the two types of searches, let us compare the functional genomic data files with video files. The ‘text search’ is like searching by keywords in the title or the description of a video file. The ‘inside data search’ is like searching for a video clip by pattern matching within the video itself. Again, it should be noted that the data formats of concern here are not DNA sequences, but rather genome-wide intensities. There is yet no software for executing the second type of searches online.

Here we present GeNemo.org, a rudimentary search engine for functional genomic data. Providing GeNemo with a bigWig or a peak file, users can search online for functional genomic data that share similar patterns at any genomic regions. Alternatively, the user can designate any online bigWig or peak file as the input to initiate the search, by providing the URL of the input file to GeNemo. The search results are reported to the user in two steps. The initial return is a synopsis of all found datasets and the corresponding genomic regions that (partially) matched with the input data. If the user clicks on an item in the synopsis, GeNemo will retrieve the specific regions of the found dataset and display it side-by-side with the user input data. Although the actual data are stored on remote servers, the current release of GeNemo offers nearly instant data retrieval and display to users. This allows the user to discover, for example, within certain genomic regions, the pattern of binding of a protein is similar to that of an epigenetic modification.

## MATERIALS AND METHODS

### Target function for search

GeNemo searches for genomic regions where the data patterns of the input data and a target dataset are similar. This search is achieved by comparing the input data to every indexed target dataset, using parallel computing. Between the input data and every target dataset, the search is based on a maximization process, which maximizes a local similarity score (*R_t_*) over the start location (*i*_1_) and the end location (*i*_2_) of a genomic region, namely:

\begin{equation*}\mathop {{\rm{arg}}\;{\rm{max}}}\limits_{t,{i_1},{i_2}} {R_t}({i_1},{i_2}),\end{equation*}

where *t* is the index of the target datasets; *i*_1_ and *i*_2_ collectively represent a genomic region, that includes a chromosome number and the start and the end positions on this chromosome. GeNemo first uses metadata to determine if a target dataset has a matched control dataset (such as an IgG ChIP-seq). If a control dataset is available, the signals of the target dataset are normalized to its respective control before calculating ${R_t}({i_1},{i_2})$.

The local similarity score is defined as:

\begin{equation*}{R_t}({i_1},{i_2}) = \log ({C_t}({i_1},{i_2}) + 1.001) + \alpha \cdot {\rm{log(|}}{i_2} - {i_1}{\rm{|)}}),\ \end{equation*}

where, ${C_t}({i_1},{i_2})$ is the Pearson correlation between the signals of the input file and the *t*_^th^_ target file between genomic coordinates *i*_1_ and *i*_2_; *α* is a tuning parameter, set at 0.01.

In the current release, GeNemo ranks all ${R_t}({i_1},{i_2})$ and outputs the datasets/regions with ${R_t}({i_1},{i_2}) >0$. If there are more than 1000 such datasets/regions, only the top 1000 datasets/regions with the largest ${R_t}({i_1},{i_2})$ are returned. We developed an algorithm to maximize ${R_t}({i_1},{i_2})$ based on Markov Chain Monte Carlo (MCMC).

### MCMC algorithm

To maximize ${R_t}({i_1},{i_2})$, we developed a MCMC method based on Metropolis–Hastings algorithm (7). We will describe the initialization, the auxiliary chain and the acceptance-rejection rule.

To initialize, our algorithm identifies ‘seed’ regions by finding all 1000-nt regions $({i_2} = {i_1} + 1000)$ on each target dataset *t* such that ${C_t}({i_1},{i_2}) \ge 0.8\;{\rm and}\;{R_t}({i_1},{i_2}) \ge 0.9$. If there are any connected regions been identified, these connected regions will be merged into one region if ${R_t}({i_1},{i_2})$ for the merged region is larger than that of each unmerged region.

We devised the auxiliary chain as follows. Denote ${i^k} = (i_1^k,\ i_2^k)$ as the boundary locations of a genomic region at step *k* of the auxiliary chain. The proposed move is to shift either boundary to either direction by up to *δ* bases. Specifically, we generate a binary random number ${A^k} \sim {\rm Binary}\ (0.5)$. If ${A^k} = 1$, the left boundary $i_1^k$ will be moved, and otherwise $i_2^k$ will be moved. Next, we generate a uniform random variable ${B^k} \sim {\rm Uniform}(1,\ 2\delta + 1)$, and then shift the corresponding boundary by $i_1^{k + 1} = i_1^k - (\delta + 1) + {B^k}$ when ${A^k} = 1$, or by $i_2^{k + 1} = i_2^k - (\delta + 1) + {B^k}$ when ${A^k} = 0$. To be theoretically complete, we set $i_1^{k + 1}$ or $i_2^{k + 1}$ as the maximum or the minimum coordinate when the boundary of a chromosome has been reached at a proposed move. Such a scenario never happens in practice because there are no functional signals in telemeric regions due to their degenerative sequence.

Our actual chain is generated by superimposing the following acceptance-rejection rule onto the auxiliary chain. The probability of accepting a proposed move is given by $\alpha = min{\rm{\ }}( {1,\frac{{{R_t}({i^{k + 1}})q({i^{\rm{k}}}|{i^{{\rm{k}} + 1}})}}{{{R_t}({i^k})q({i^{{\rm{k}} + 1}}|{i^{\rm{k}}})}}} )$, where ${R_t}({i^k}) = {R_t}(i_1^k,\ i_2^k)$, and $q({i^{{\rm{k}} + 1}}|{i^{\rm{k}}})$ is the probability of moving from $(i_1^k,\ i_2^k)$ to $(i_1^{k + 1},\ i_2^{k + 1})$ in the auxiliary chain. Given how the auxiliary chain is constructed, we derive that $q({i^{{\rm{k}} + 1}}{\rm{|}}{i^{\rm{k}}}) = \frac{1}{2} \cdot \frac{u}{{2*\delta + 1}}$, where $u = 1$ when chromosome boundaries are not reached. To be theoretically complete, when a chromosome boundary is reached, $u = 1 + {u^*}$ and ${u^*}$ is the number of bases beyond the boundary. We recall that in this case the auxiliary chain puts ${i^{{\rm{k}} + 1}}$ on the boundary coordinate of the chromosome. It is easy to see that our actual chain is ergodic and reversible.

### Parallel computing

To minimize response time, the current release of GeNemo creates up to 24 parallel computing threads for every search. The target datasets are separated into up to 100 non-overlapping subsets, and each subset is handled by a computing thread. The results of these computing threads, in the form of ${R_t}({i_1},{i_2})$, are assembled and then ranked. We tested a series input files using 1, 10 and 20 computing threads, and found that 20 threads typically accelerated the search by 7–9 folds (Supplementary Table S4).

### Replacing signals from real data by noise for simulation

In order to make a ‘negative’ region to contain a noise profile (the shape of intensities in this region) that appears similar to ‘positive’ regions, we generated the noises in two steps. In step 1, the noise locations were generated on every chromosome by a Poisson process, $BP({N_i},\lambda )$, where *N_i_* is the total number of bases of Chromosome *i*, and $\lambda = 0.01$. In step 2, on every noise location, a noise value was generated by a Gaussian distribution $G(\mu ,{\sigma ^2})$, where *μ* and *σ*^2^ were given by the mean and the variance of the intensities of all the positive regions. We note that the noise locations should be close to each other, because the interval between two consecutive noise locations should follow an exponential distribution ${\rm Exp}(\lambda = 0.01)$.

## RESULTS

### The current release of GeNemo search engine

The current release of GeNemo can search against all the released functional genomic datasets produced by the ENCODE and mouse ENCODE projects. As of September 30, 2015, there were a total of 3312 datasets from histone and transcription factor ChIP-seq, DNase-seq and FAIRE-seq experiments. These datasets are located on remote data servers and are not managed by GeNemo developers. We developed a program to index these remote datasets and create a metadata on the GeNemo server. Anticipating future data releases, we will use this program to index additional datasets and thus expand the pool of target files.

**Figure 1.**
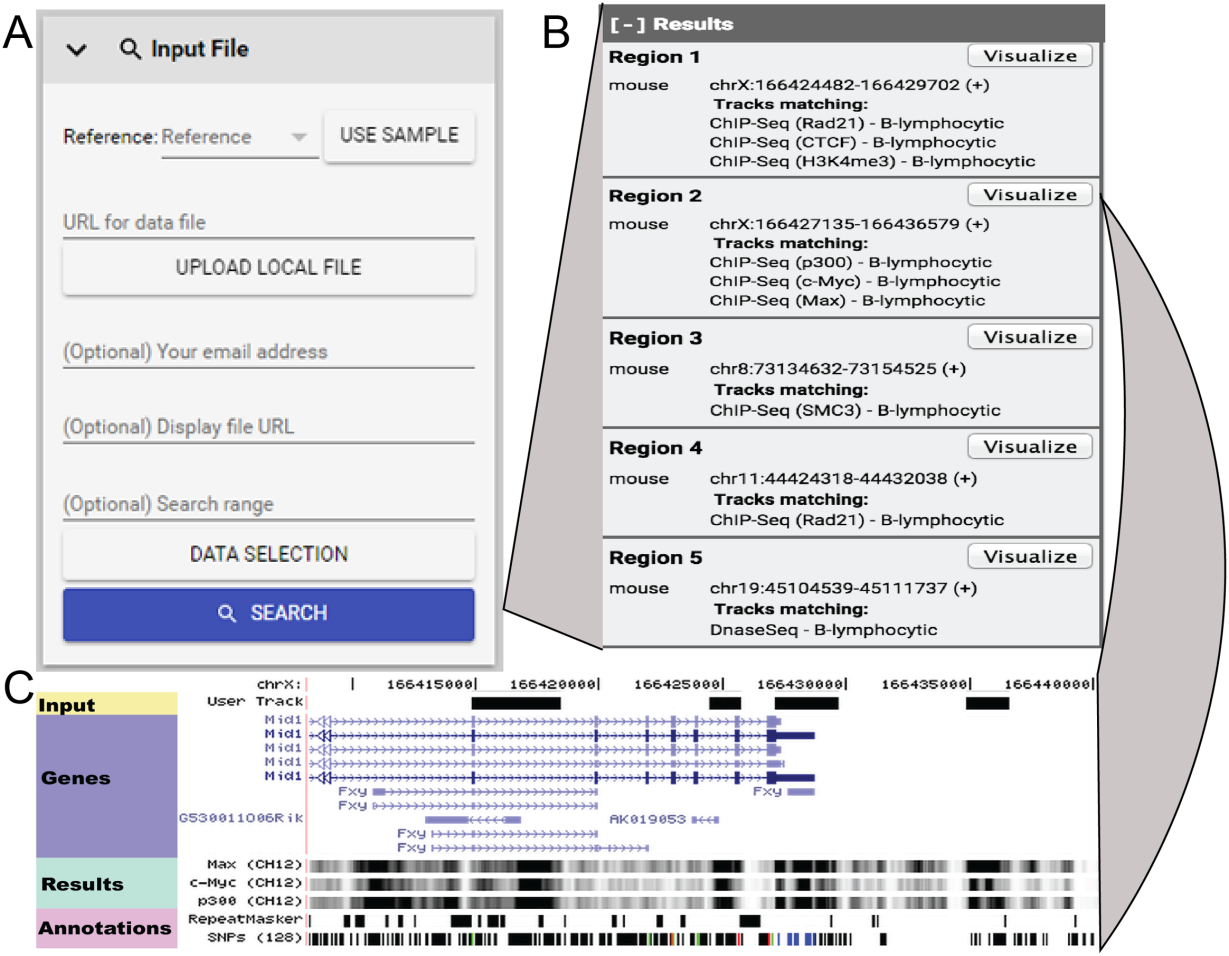
GeNemo screens. (**A**) Input screen, references and the data file is needed to search; (**B**) Results screen, the coordinates and tracks matching are shown; (**C**) Visualization screen, the input track, genes, matching tracks (shown as results) and other annotations are shown.

GeNemo has a simple user interface (Figure 1A). The minimum required input from the user is a bigWig file or a peak file in BED format and choice of the species. The user can either upload the input file from her/his own computer, or designate a URL if her/his file is online. GeNemo will automatically detect the file type. With the input files, the user can click the search button to initiate the search. The user can also click the ‘use sample file’ button to supply GeNemo with the sample input file.

The more sophisticated users can take advantage of a few optional inputs. The user can supply an email address. In this case, GeNemo will send an email with a link to the search results. This is useful when the user wants to keep a record of the search outcome, or the search takes more than a few minutes and that is beyond the user's patience.

The user can choose what datasets to search against, by clicking the ‘data selection’ button. For example, she/he can choose to search against the entire collection of ENCODE datasets, by clicking on ‘Use filters -> Select all -> Add’. Alternatively, she/he can constraint the search to any subset of the target files. For example, the user can constraint the search space to certain cell types or certain kinds of experiments.

GeNemo returns the search results typically in minutes. The results are displayed in user's web browser. In addition, a web link to the results is sent to user, if an email address was provided. GeNemo reports the search results in two steps. The initial return is a synopsis of all found datasets (Figure 1B). Each entry reports a genomic region where a found dataset exhibited similar patterns to the input. The user can further investigate a found dataset by clicking the ‘Visualize’ button next to each entry. This is an intuitive design (imagine clicking on a Google found entry to browse the found website). This action will invoke a genome browser-like display (3). The user input data will be displayed in parallel to the found data, centered at the genomic region where a pattern match was found (Figure 1C). The user can navigate these datasets by zooming and shifting her/his genomic views using the ‘Navigation’ panel. Even though the found data are stored on remote data servers that are not managed by GeNemo developers, we optimized the data retrieval strategy such that the user would not experience noticeable waiting time for data visualization. GeNemo instantly displays the input and the found data, even when the user navigates to other genomic regions.

### Simulation analysis

We carried out three simulation studies to test algorithm performance.

In the first study, we simulated the cases where all the genomic regions with matched signals were completely known (gold standard was available). We generated three datasets. Each dataset was composed of one input file and one target file. Both simulated files covered the entire length of the human genome. Each pair of files contained certain number of regions with matched signals, termed ‘matched regions’ hereafter (Supplementary Table S1). Each matched region contained a random number of signal segments (between 1 and 10), and each signal segment had a random length (between 100 and 2000 bp). These matched regions were inserted to the simulated genome at random positions. The input and the target files had the same matched regions at the same locations. For the rest of the genome, we inserted additional signal segments at random locations to the target files, and kept the input file free of any additional randomly inserted signals. These three pairs of input and target files were subjected to the search. Consistent to the simplicity of the simulated data, all the matched regions in datasets 1 and 2 were found; the algorithm did not report any additional matched regions (Supplementary Table S1). In dataset 3, the algorithm only reported 1000 of the 1500 matched regions. This was expected because our program was set to only output the top 1000 matches.

The second simulation was carried out in a similar fashion, except that additional signal segments were randomly added to both the input and the target files (Supplementary Table S2). This is to better mimic the noises in actual experiments and the errors in data processing. The caveat of this simulation is that it does not offer a ‘gold standard’ test dataset. This is because if there were two additional signal segments with overlaps in the input and the target files, they should be considered as a match in the context of a pattern search, even though they were outside of the designated ‘matched regions’. If we consider two random typos that happened to match each other, from the perspective of a text search, it would be correct to find a match between the two typos. In evaluating the algorithm, we chose not to include the overlaps of the randomly inserted additional signals into the ‘true positive’ set. We recognize that this choice would lead to underestimation of the precision of the algorithm. Still, the algorithm found the majority of the matched regions with high precision (Supplementary Table S2).

The third simulation utilized a real dataset (H3K27ac ChIP-seq in Bruce4 ES cells) to generate the input file. First, to obtain the ‘positive’ match regions, we searched this dataset (wgEncodeEM002497) against itself and obtained the top 100 matched regions. These regions were regarded as positive regions. The rest of the genome was regarded as ‘negative’ regions. Signals within these positive regions were kept, and signals on the rest of genome were replaced by noises (see ‘Materials and Methods’ section). This produced a synthetic dataset (Dataset 7, Supplementary Table S3). We used this synthetic dataset to search against all ENCODE datasets. We obtained top 100 GeNemo returned regions. We regarded a returned region as ‘true positive’ if this match was generated from the original wgEncodeEM002497 track and it overlapped with a positive region. Next, we repeated this simulation by first obtaining 500 and 1000 ‘positive’ regions (Datasets 6 and 7, Supplementary Table S3). GeNemo's sensitivity and precision in these simulations ranged from 100% to 89.9% (Supplementary Table S3). The search took ∼10% more time in Dataset 9, where the positive regions covered ∼1.3% of the effective genome size, than those (Datasets 6 and 7) with smaller positive regions (Running time, Supplementary Table S3).

### Data applications

**Figure 2.**
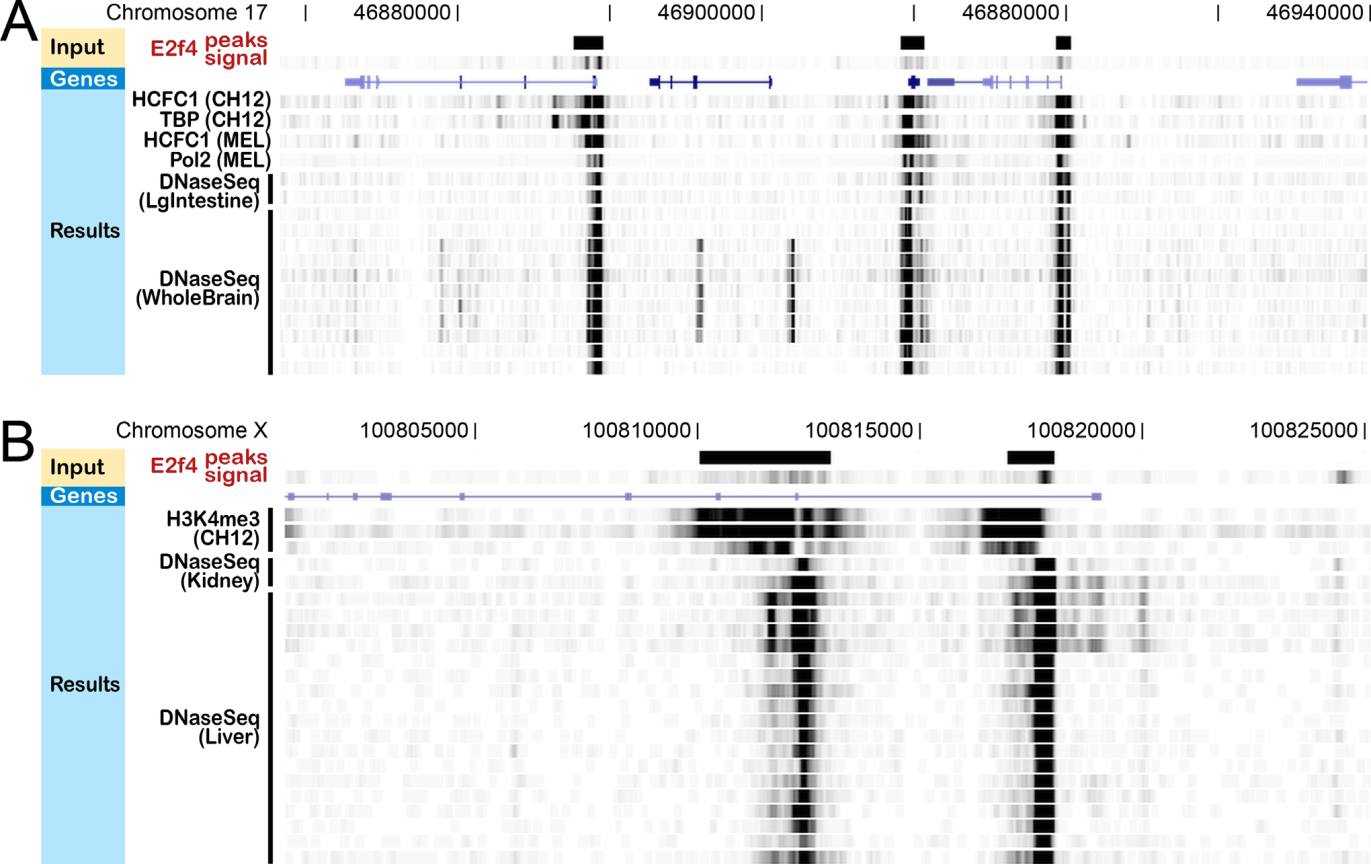
Results for E2F4 ChIP. (**A**) Result in chromosome 17. Several other ChIP experiments (HCFC1, TBP, Pol2) and DNase-Seq experiments in large intestine (LgIntestine) and whole brain (WholeBrain) are shown to have similar signals around this region. (**B**) Result in chromosome X. ChIP experiments for epigenetic signal (H3K4me3) and DNase-Seq experiments in kidney and liver are shown to have similar signals.

We will present a data example. We retrieved a public dataset from the GeNemo indexed files. This is a bigWig file for E2F4 ChIP-seq in B-cell Lymphoma (CH12) (8). We used this bigWig file as the input, and used the ‘data selection’ tool to specify GeNemo to search against all the ENCODE datasets other than E2F4 ChIP-seq experiments. GeNemo returned 1000 similarity regions (Supplementary Figure S1). Some top ranked regions suggested co-binding of E2F4 with the core transcriptional machinery such as TBP and Pol2 in lymphoma (CH12 tracks, Figure 2A) and leukemia (MEL tracks, Figure 2A). Some of these regions were precisely decorated with H3K4me3, an epigenetic mark associated with transcriptional activation (CH12 tracks, Figure 2B) or bivalent domains (9). Although E2F4 was generally considered a transcriptional repressor, these data suggest that E2F4 may also contribute to transcriptional activation or bivalent regulation in blood cancers. Consistent to this idea, overexpression of E2F4 and its transcriptional cofactors led to both transcriptional repression and activation in lymphoblastoid cell lines (10).

We did not expect the same E2F4 bound regions mentioned above to match DNA hypersensitivity regions in normal tissues, including brain (WholeBrain tracks, Figure 2A), large intestine (LgIntestine tracks), kidney (Kidney tracks, Figure 2B) and liver (Liver tracks, Figure 2B). This makes us to posit that the identified regions are regulatory sequences used for transcriptional control in normal tissues; some blood cancers potentially used these regulatory sequences for transcriptional activation, which were attached with E2F4. This example illustrates that GeNemo may be used as a hypothesis generating tool. However, we recognize the gap between any hypotheses generated by association and functional validation.

## DISCUSSION

We anticipate that online search engines will revolutionize the utilities of biomedical big data, like Google did with text big data. However, except for medical records, many types of biomedical data cannot be searched as text. Functional genomic data are a point in case. Despite their increasing importance to biomedical research, to our knowledge, there is yet no online search engine for them. We note that the search methods for genomic sequences (text based or string based) are very different from, and probably irrelevant to the searches for functional genomic data. The latter represents the extent of molecular activities at every genomic location. Therefore, it requires new computational engineering efforts that are customized to this data type.

GeNemo is the first online search engine for functional genomic data. Many aspects of the design and the implementation of this search engine were made to optimize the speed. This is to offer the users the opportunity to ‘interact’ with the search engine by executing more than one search. We anticipate GeNemo to release the research power of physicians and biologists at large. No programming or bioinformatic expertise is required. GeNemo reduces months of work on data processing and computation into minutes.

The price to pay for the flexibility and scalability of an online search engine is that the raw datasets cannot be downloaded and processed at a centralized data repository. This makes a number of normalization techniques not applicable. The current release of GeNemo has a limited number of target datasets. To expand the target datasets, we developed programs to index additional datasets and expand GeNemo's metadata. However, the next round of expansion on indexed datasets requires substantial investments on hardware, software and engineering fronts. Speed enhancements would be another important future direction. These efforts should probably be undertaken as industrial goals.

## Supplementary Material

SUPPLEMENTARY DATA
