# Supplementary material for: GeNemo: a search engine for web-based functional genomic data
Source: Nucleic Acids Res. 2016 Apr 20;44(Web Server issue):W122–7. doi: 10.1093/nar/gkw299 (PMC4987887; doi:10.1093/nar/gkw299)
Supplement: SUPPLEMENTARY DATA [file supp_44_W1_W122__index.html]

GeNemo: a search engine for web-based functional genomic data — GeNemo: a search engine for web-based functional genomic data — SUPPLEMENTARY DATA 

# GeNemo: a search engine for web-based functional genomic data

## SUPPLEMENTARY DATA

- SUPPLEMENTARY DATA
