# Supplementary material for: GeNemo: a search engine for web-based functional genomic data
Source: Nucleic Acids Res. 2016 Apr 20;44(Web Server issue):W122–7. doi: 10.1093/nar/gkw299 (PMC4987887; doi:10.1093/nar/gkw299)
Supplement: SUPPLEMENTARY DATA [file supp_gkw299_nar-00300-web-b-2016-File002.docx]

**Supplementary Figures**

Figure S1. Mean R value of the E2F4 ChIP-seq search. The mean of R values for given number of top regions in the results (up to 1000).

**Supplementary Tables**

Table S1. GeNemo results for Simulation Set #1.

|  | # of regions with matched signals | # of signal-containing segments per matched region | Average length of signal-containing regions | Sensitivity | Precision |
| --- | --- | --- | --- | --- | --- |
| Dataset 1 | 100 | 1 – 5 | 500 | 100% | 100% |
| Dataset 2 | 500 | 1 – 10 | 1,000 | 100% | 100% |
| Dataset 3 | 1,500 | 1 – 10 | 1,500 | 66.7% | 100% |

Table S2. GeNemo results for Simulation Set #2.

|  | # of regions with matched signals | # of signal-containing segments per matched region | # of additional signal segments | Sensitivity | Precision |
| --- | --- | --- | --- | --- | --- |
| Dataset 4 | 100 | 1 – 5 | 200 | 100% | 89.1% |
| Dataset 5 | 500 | 1 – 10 | 1,000 | 100% | 86.6% |
| Dataset 6 | 1,500 | 1 – 10 | 3,000 | 68.4% | 100% |

Table S3. GeNemo results for Simulation Set #3.

|  | # of positive regions | Total length of positive regions (bp) | # of true positives | Sensitivity | Precision | Running time (s) |
| --- | --- | --- | --- | --- | --- | --- |
| Dataset 7 | 100 | 6,935,500 | 100 | 100% | 100% | 2,147 |
| Dataset 8 | 500 | 18,008,700 | 500 | 100% | 100% | 2,150 |
| Dataset 9 | 1,000 | 25,737,500 | 898 | 89.8% | 89.8% | 2,330 |

Table S4. Computational time with different number of threads in use.

| **Ref.** | **Track name** | **Cell type** | **Antibody** | **Computational time with # of threads (s)** | | |
| --- | --- | --- | --- | --- | --- | --- |
|  |  |  |  | **1** | **10** | **20** |
| mm9 | wgEncodeEM001937 | CH12 | E2F4 | 552 | 81 | 60 |
| mm9 | wgEncodeEM001954 | CH12 | CTCF | 1,386 | 249 | 185 |
| hg19 | wgEncodeEH001014 | HeLa-S3 | H3K27ac | 914 | 140 | 112 |
| hg19 | wgEncodeEH000033 | GM12878 | H3K4me1 | 1,013 | 178 | 134 |

Table S5. Number of experimental datasets released by ENCODE and ENCODE mouse projects. Each experiment may include 1 – 3 biological replicates.

| Species | Data Type | # of Cell Types | # of Antibodies | # of Experiments |
| --- | --- | --- | --- | --- |
| Human | DNase-Seq | 180 | N/A | 394 |
|  | RNA-Seq | 51 | N/A | 455 |
|  | Histone | 69 | 48 | 486 |
|  | TFBS | 93 | 187 | 1274 |
| Mouse | DNase-Seq | 44 | N/A | 149 |
|  | Histone | 28 | 12 | 159 |
|  | TFBS | 25 | 46 | 167 |
|  | RNA-Seq | 53 | N/A | 185 |
